# The advantage of 3D conformal treatment of lumbar spine metastases in comparison to traditional PA or AP-PA techniques: restoring an intermediate niche of therapeutic sophistication

**DOI:** 10.1186/1748-717X-8-34

**Published:** 2013-02-12

**Authors:** Viacheslav Soyfer, Benjamin W Corn, Natan Shtraus, Dan Schifter, Haim Tempelhof

**Affiliations:** 1 Radiation Oncology Department, Tel Aviv Souraskiy Medical Center, affiliated with Tel Aviv University School of Medicine, 6 Weizmann St., Tel Aviv, 64239, Israel

**Keywords:** Spine, Metastases, Radiation, IMRT, 3D

## Abstract

**Background:**

To evaluate the effect of the 3D radiation field design on normal tissues compared with commonly used appositional fields in patients with lumbar spine metastases.

**Methods and materials:**

Ten comparative treatment plans for radiation of lumbar spine metastases were compared for posterior and anterior- posterior fields with 3D plans.

**Results:**

The PTV coverage in all comparative plans was similar. V 15 of the bowel in 3D, AP-PA and PA plans was 6.7 Gy (SD 6.47), 39.8 Gy (SD 11.4) and 37.3 Gy (SD15.7), respectively (p < 0.0001). The mean dose to both kidneys was 9.6 Gy (SD 4.8), 4.1 Gy (SD 3.9) and 4.6 Gy (SD 4.4) for appropriate plans (p = 0.002). Maximal dose to the spinal cord was 30.6 Gy (SD 2.1), 33.1 Gy (SD 9.8) and 37.7 Gy (SD 2) for 3D, AP-PA and PA plans.

**Conclusion:**

3D conformal treatment planning of lumbar vertebral metastases was significantly better in term of bowel and spinal cord exposure compared to AP-PA and PA techniques. The exposure of the kidneys in 3D plans, while greater than in the comparative plans, did not violate accepted dose-volume thresholds.

## Introduction

Bone metastases constitute a growing oncologic problem due to increasing life expectancy among cancer patients [1]. The spine remains one of the most frequently involved sites for metastatic disease. In one study that evaluated 832 patients who died of their cancer, vertebral involvement was identified in 36% at autopsy [2].

One of the most commonly used approaches to treat painful bone metastases is external beam radiation therapy. The efficacy of radiation therapy in terms of pain alleviation ranges between 50–80% [3]. Multiple publications including randomized studies, meta-analyses and guidelines have reflected on the best dose and fractionation needed to achieve durable palliation. The most popular regimens are 30 Gy in 10 fractions or 20 Gy in five fractions. A single 8 Gy fraction seems to be as effective as more protracted schedules [4-6].

Traditional technique for treating metastases of the spine is titrated to the location of the lesions. In the lumbar region, typical fields include a single posterior beam or anterior-posterior (AP-PA) portals, usually depending on the skin- target distance.

Over the past several decades the external beam irradiation armamentarium has expanded to include conformal three - dimensional (3D) radiotherapy, intensity modified radiation therapy (IMRT) and stereotactic approaches. Although these methods have been readily adopted by senior clinicians [7], it is difficult to identify peer-reviewed articles discussing the advantages of these new techniques above classical AP-PA or single posterior-anterior (PA) beam treatment of vertebral column metastases. Andic et al. analyzed three-dimensional (3D) data of conventional two- dimensional (2D) palliative spinal bone irradiation using different reference points and treatment plans with respect to the International Commission on Radiation Units and Measurements (ISRU) Report 50. They concluded that in palliative spinal bone irradiation, 2D conventional single posterior field radiotherapy did not comply with the ICRU Report 50 recommendations for PTV dose distribution, while AP-PA field plans did attain the intended dose ranges with a homogenous distribution and reasonable doses to the medulla spinalis, esophagus and intestines [8] However, this relatively recently published study did not investigate the multiple field arrangements that might further improve the conformality of treatment Accordingly we were interested in assessing the relative benefits of several straightforward external beam approaches for treating spinal metastases with respect to target volume coverage and the potential influence of these respective beam arrangements on normal tissue (e.g., kidneys, small bowel, and spinal cord) tolerance.

Despite the fact that formal comparisons of 3-D techniques with even more traditional techniques (i.e., PA only, AP-PA) could have been carried out years ago, the absence of such comparisons in the literature prompted us to rigorously evaluate this matter. The issue has assumed greater importance because today, patients with bone metastases live longer [1] and therefore the opportunity to manifest delayed complications has become greater. Moreover, hypofractionated regimes are often considered in an era where not only stereotactic body radiation therapy but also intensity modulated radiation therapy is practiced. As such, large doses are given to associated organs per fraction with heightened concerns for late toxicity.

## Methods and materials

Patients’ characteristics are presented in Table 1.

**Table 1:** Patients’ characteristics

**Treated vertebrae**	**Diagnosis**	**Gender**	**Age**	**Number**
L2-L4	Carcinoid	Female	60	1
L2-L5	Lung Ca	Male	77	2
D10-L2	Lung Ca	Male	48	3
D10-L2	Breast Ca	Female	44	4
L1-L3	Prostate Ca	Male	83	5
L1-L5	Breast Ca	Female	70	6
L1-L3	Prostate Ca	Male	76	7
L3-L5	Lung ca	Male	77	8
L1-L4	Prostate Ca	Male	87	9
L1-S1	Lung	Male	71	10

The table depicts patients’ demographic characteristics.

Ten comparative plans were evaluated from randomly chosen patients with lumbar spine metastases who were treated from 2007 to 2010. Ages ranged from 44–86 (median 74). There were 4 women and six men. The distribution of primary tumors included three patients with lung cancer, three men with prostate cancer, two women with breast cancer, one patient with colorectal cancer as well as one individual with gastric cancer.

### Planning approaches

Every patient underwent CT simulation on a Big Bore CT unit (Philips, Eindhoven, Netherlands) with 3 mm slices. All patients were placed in the supine position with arms above the head. The gross tumor volume (GTV) was delineated in accordance with the visible lesions on imaging studies. A normal appearing vertebral body above and below the radiographic abnormality was encompassed to generate the clinical treatment volume (CTV). The planning treatment volume (PTV) included an additional 5–10 mm around the CTV in deference to multi-leaf collimator constrants and daily position inaccuracy. The upper and lower margins of PTV were limited at the intervertebral disc spaces. Critical organs (kidneys, spinal cord and bowel) were delineated separately. Bowel loops were easily identified on each slide rather than delineating the whole abdominal cavity. Patients were immobilized using a standard laser system. Total doses of 30 Gy in 3 Gy fractions were prescribed to the point located at the center of the PTV that is in accordance with the ICRU 50. The energy of X rays was 6 or 18 MV or their combinations depending on the depth of the isocenter.

**Figure 1:**
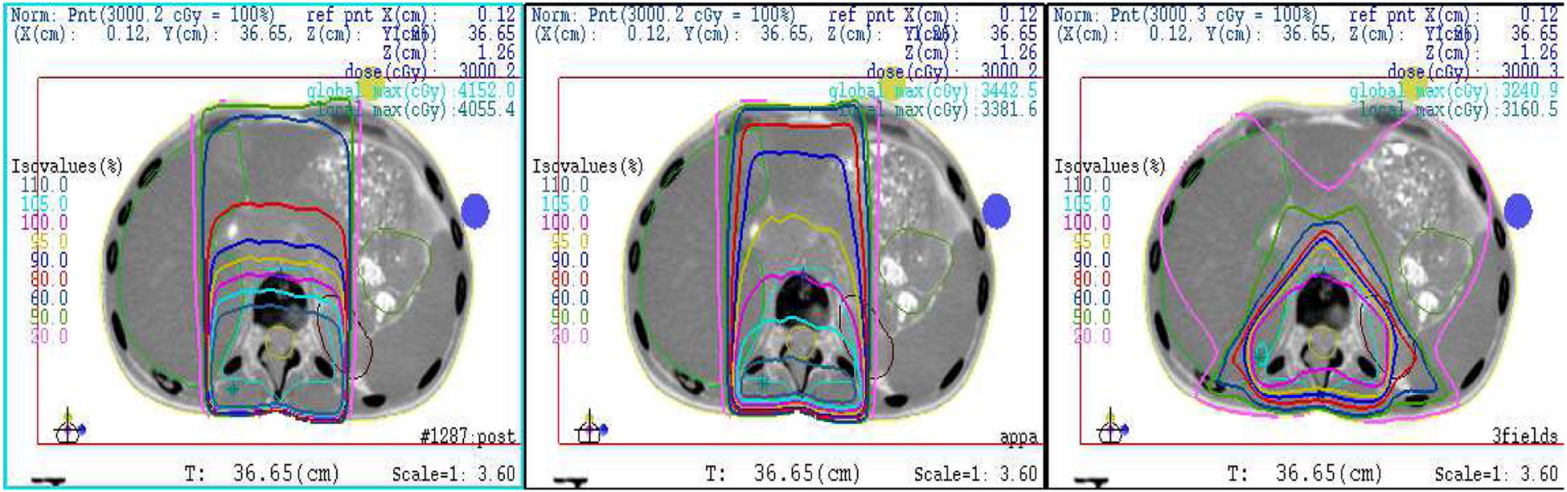
**PA, AP-PA and 3D conformal plans for a typical patient.** Three representative plans are displayed in axial view for 3D conformal, AP-PA and PA beams technique.

A series of plans (i.e., PA, AP-PA and 3D) was assessed for each patient. Representation of 3D conformal, AP-PA and PA plans for a typical patient is depicted in Figure 1. The three-dimensional conformal plan consisted of 2 posterior wedged oblique beams of 120–140 and 220–240 degrees on each side as well as one posterior beam. The data were collected for CTV coverage, mean dose and V15 exposure of the bowel, mean dose for both kidneys and maximal dose of the spinal cord for every patient in all comparative plans. All above mentioned parameters were calculated for their mean values for the patients in the study. The best available plan was selected for treatment. Verification set up films were taken prior to the initiation of treatment.

### Statistical considerations

A comparison of group means of all the parameters was performed using a one-way analysis of variance. The Multiple comparison adjustment method “GT2” by Hochberg [9] was employed to determine significant differences between pairs of groups. SAS for Windows version 9.2 was used for the analysis.

## Results

**Figure 2:**
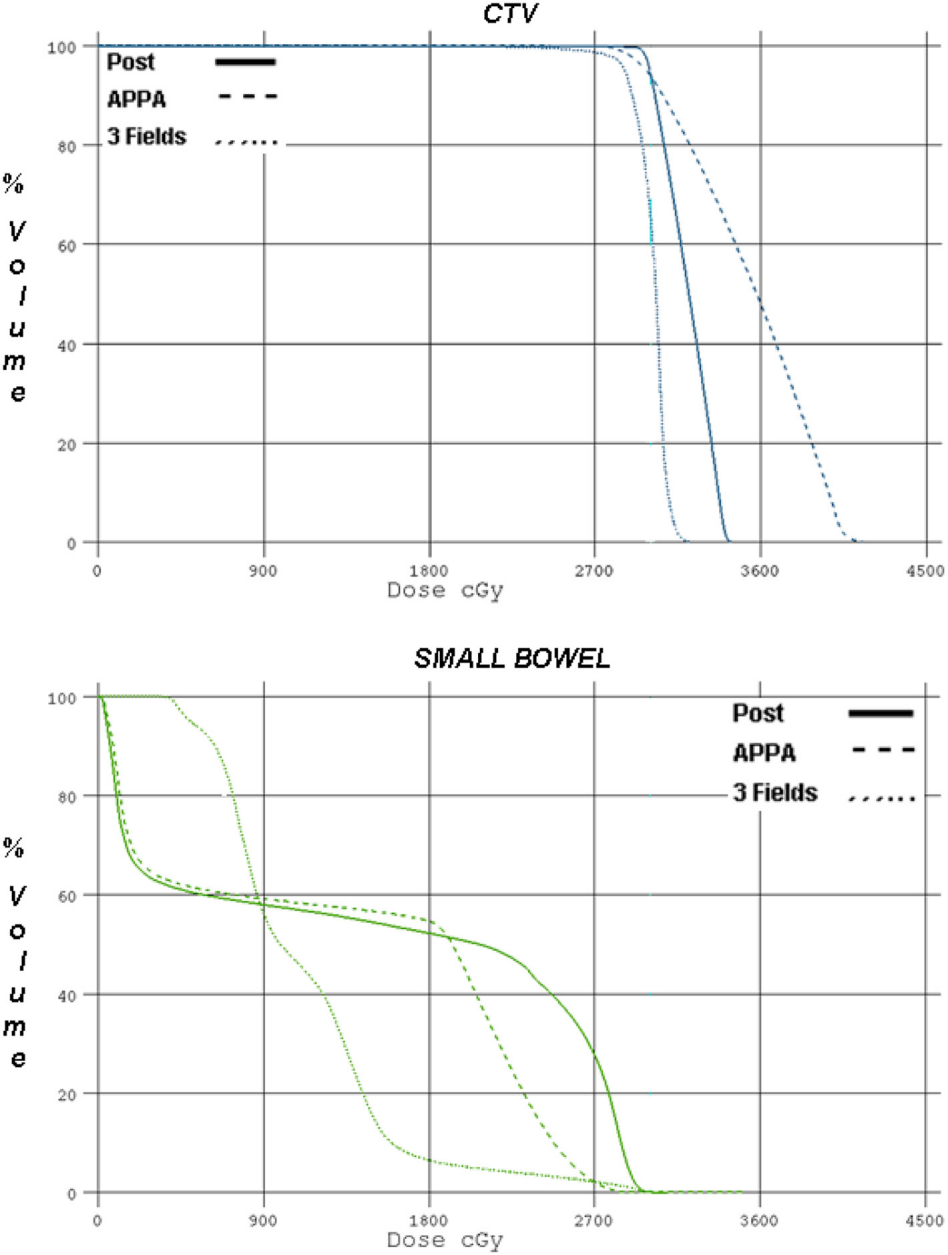
**Dose volume histograms for PTV and bowel.** It can be seen that the CTV coverage is similar for all 3 plans while there is improved bowel sparing in the 3D plan.

**Figure 3:**
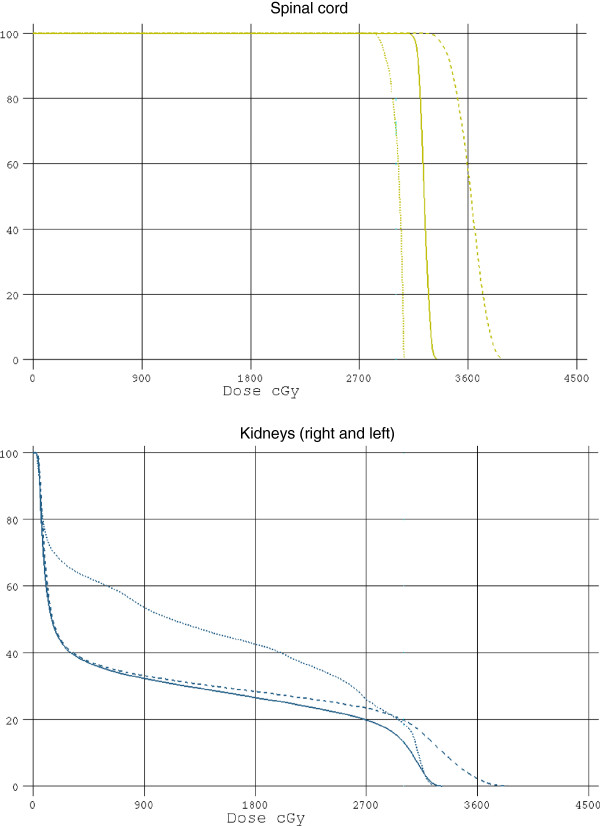
**Dose volume histogram of the spinal cord and kidneys.** It is evident that the 3D plan offers improvement in maximal dose deposited to the spinal cord while maintaining the kidney dose within tolerable limits.

**Figure 4:**
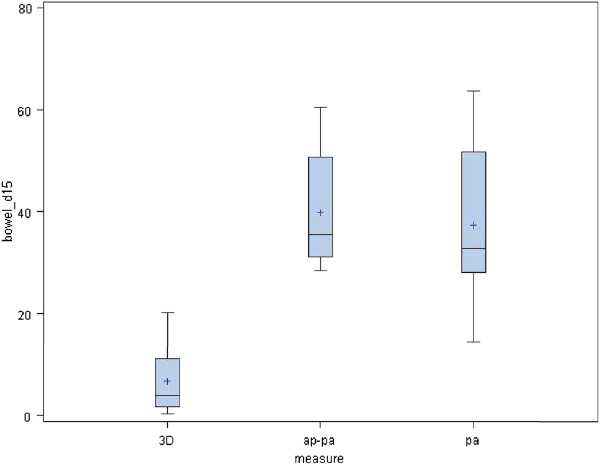
**V 15 of the bowel in 3D, AP-PA and PA plans.** These histograms show that the best V15 is achievable with the 3D conformal plan.

**Figure 5:**
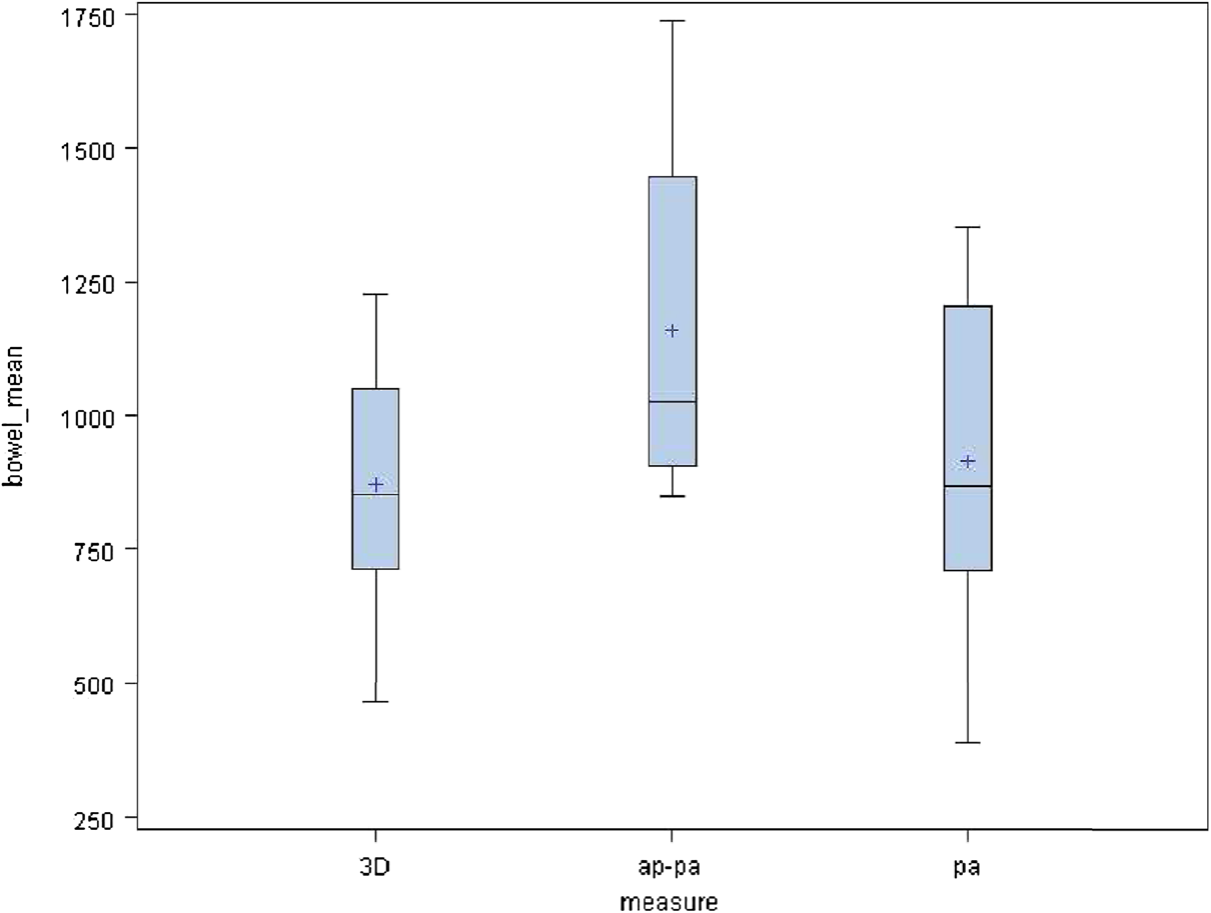
**Mean dose to the bowel.** These histograms show that the best mean dose to the bowel is associated with the 3D conformal plan.

**Figure 6:**
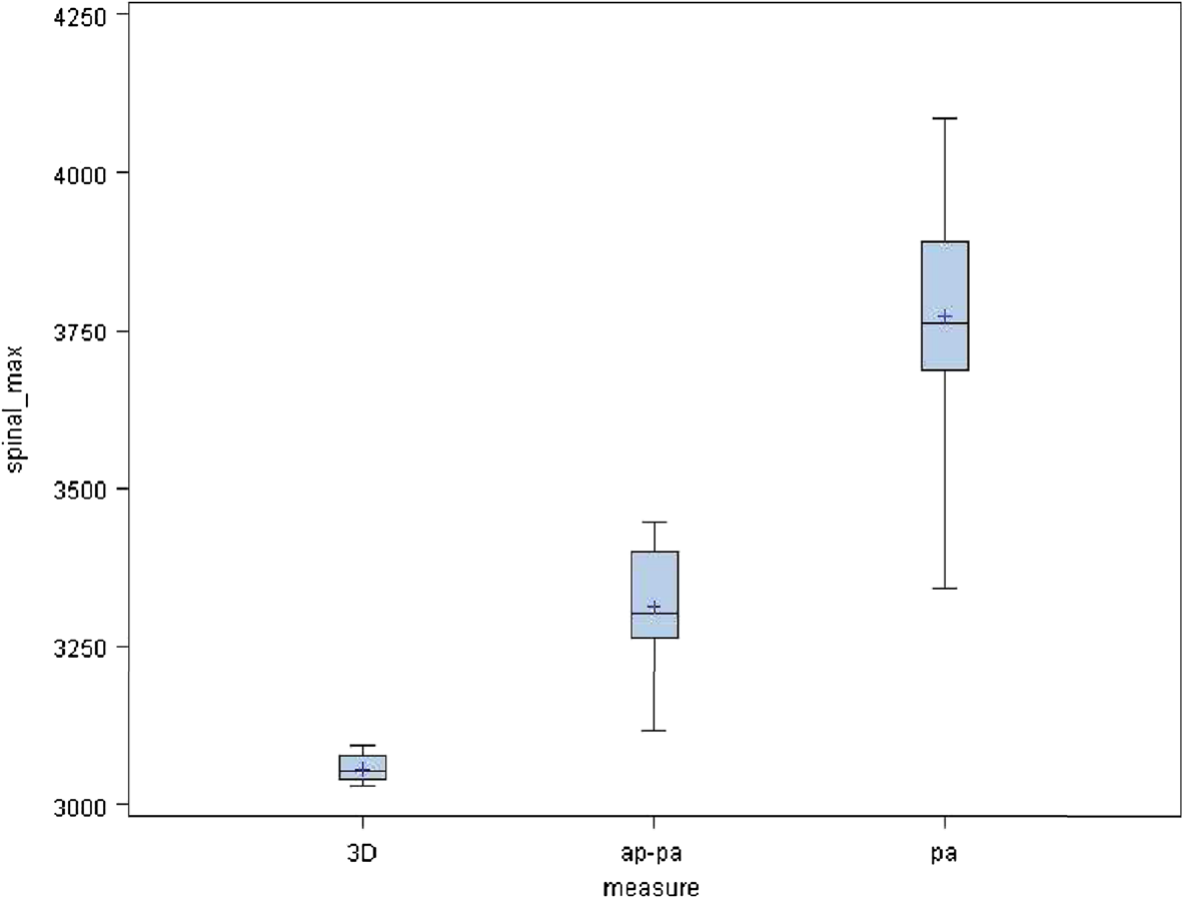
**Maximal dose to the spinal cord.** These histograms show that the lowest maximal spinal cord dose is achievable with the 3D conformal plan.

Three representative plans are displayed in axial view for 3D conformal, AP-PA and PA beams technique (Figure 1). Dose volume histograms for CTV and bowel are represented in Figure 2. It can be seen that the CTV coverage is similar for all 3 plans while there is improved bowel sparing in the 3D plan. 95% of the prescribed dose of 30 Gy in 10 fractions covered the CTV in all comparative plans. Dose variations between + 7% and –5% were not exceeded in any plan and hot spot above these limits were not allowed. DVH of the spinal cord and kidneys is represented in Figure 3. It is evident that the 3D plan offers improvement in maximal dose deposited to the spinal cord while maintaining the kidney dose within tolerable limits. The V 15 of the bowel in 3D, AP-PA and PA plans were as follow 6.7% (SD 6.47), 39.8% (SD 11.4) and 37.3% (SD15.7), respectively (p < 0.0001). (Figure 4) These histograms show that the best V15 is achievable with the 3D conformal plan. Mean dose to the bowel was 8.7 Gy (SD 2.2), 11.6 Gy (SD 3.2) and 9.2 Gy (SD 3) (p < 0.0003) for the respective beam arrangements (Figure 5). These histograms show that the best mean dose to the bowel is associated with the 3D conformal plan. The mean dose to both kidneys was 9.6 Gy (SD 4.8), 4.1 Gy (SD 3.9) and 4.6 Gy (SD 4.4) for these respective plans (p = 0.009). The maximal dose to the spinal cord was 30.6 Gy (SD 2.1), 33.1 Gy (SD 9.8) and 37.7 Gy (SD 2) for 3D, AP-PA and PA plans, respectively (Figure 6). These histograms show that the lowest maximal spinal cord dose is achievable with the 3D conformal plan. The radiation exposure to critical organs is presented in Additional file 1.

## Discussion

Irradiation has constituted a traditional therapeutic option for palliation of lumbar metastases. Historically, extensive research has explored optimal dose-fractionation relationships for his modality. However, despite the modern revolution in treatment planning there is limited information on sophisticated field configuration for the irradiation of the lumbar spine. Our results indicate that similar PTV coverage can be achieved in the three comparative plans selected but that the optimal dose distribution is associated with the 3-D plan which was developed.

Approximately 70% of cancer patients have metastatic disease at death. The spine is involved in up to 40% of those patients. Spinal cord compression may develop in 5% to 10% of cancer patients and up to 40% of patients with preexisting nonspinal bone metastasis (>25,000 cases/y). Given the increasing survival times of patients with cancer, greater numbers of patients are likely to develop this complication [10]. Such lesions are most frequently encountered in the management of patients diagnosed with cancers of the breast and prostate. Patients with bone metastases that arise from the latter two primary malignancies can enjoy median survival times that are measured in years while mean survival times as low as 6 months from bronchogenic carcinoma are seen in contemporary series [11].

External beam irradiation constitutes a time-honored intervention for the treatment of bone metastases; especially those situated in the spine. Controversies surrounding dose fractionation schedules for bone metastases have preoccupied radiation oncologists for years [12]. Not only retrospective series but also numerous randomized controlled clinical trials have stated that similar pain relief outcomes are achievable with short and long course of radiotherapy. Prescriptions for radiation treatment, however, require more specification than the delineation of dosage, beam energy and notation of total and fractional dose.

Despite the abundance of information regarding dose-fractionation regimens for spinal metastases, limited data exist regarding the comparison of various techniques in the treatment of the spine [13]. Although sophisticated approaches such as stereotactic body irradiation for spinal metastases have been readily adopted by many institutions [14] such technologies are not available to all users around the world. Therefore, a formal assessment must be made of more accessible technologies.

Radiation oncologists have always concerned themselves with both components that comprise the “therapeutic index”. Our data underscore the value of the 3D conformal approach for spinal metastases as a palliative tool. When treating the lumbar spine; however, several organs are at a risk for expressing radiation-related damage. In term of anticipated acute small bowel toxicity Baglan et al. showed that irradiation of more than 15 Gy to at least 150 cm3 is associated with an incidence of grade 3 acute small bowel toxicity approximating 30% using the Common Toxicity Criteria scale [15,16]. The mean radiation dose at which diarrhea grade 2–3 occurred was 27 Gy according to Gunnlaugsson A et al. [17]. Comprehensive review of the radiation dose- volume effects in small bowel was published by Kavanagh et al. in 2010 [18]. According to those authors, following doses on the order of 50 Gy, late small-bowel obstruction or perforation rates of 2% to 9% were observed after partial organ irradiation. A dose of 25 Gy in five fractions of preoperative radiation therapy is associated with the same rate of late toxicity. It was also underscored in the review that small bowel obstruction occurred in 30% when fields were extended to the level of L1 or L2 versus 9% with pelvic- only therapeutic strategies [16]. Reports on the probability of the late small bowel damage could not be identified in correlation with the radiation dose- volume function. Notwithstanding, we reasoned that maximum effort should be invested to spare as much small bowel from the radiation field as possible. As a surrogate, we reported in our study the mean volume of the bowel irradiated and V15.

It must be acknowledged that one of the possible disadvantages in conformal 3D radiation techniques which employ a paired set of oblique wedged fields is the deposition of higher doses within the kidneys. The risks of radiation damage to the kidneys were comprehensively summarized by Dawson et al. The dose associated with a 5% risk for toxicity at 5 years was 18–23 Gy regardless of the fractionation scheme used [19]. It was within the context of these criteria that we carried out a comparative assessment of potential renal damage from radiation treatment.

Although the beam arrangements evaluated herein were not associated with optimal renal sparing, the dose volume histograms which paid attention to the kidney suggest that such treatments can be delivered without inducing nephropathy [18]. Moreover, vis-à-vis other adjacent critical organs (e.g., bowel, spinal cord) the conformal approach offered significant improvement. As indicated, none of the 3 approaches evaluated was associated with advantages in terms of target coverage.

A recently published trial by the RTOG (97–14) assessed quality of life endpoints among patients suffering from bone metastases [20,21]. As predicted, radiation therapy was highly effective in reducing bone pain. The framers of the protocol allowed physicians to treat patients who presented with spinal metastases via AP-PA or posterior-only portals. Despite the availability of conformal approaches during the recruitment period of the protocol, no allowance was made for selecting such an option. While this decision may have successfully isolated the dose fractionation question (i.e., by eliminating the variability in radiation treatment approaches) it is possible that patients enrolled in the study were impacted upon from a QOL perspective due to the use of non-optimized portal arrangements.

The RTOG is about to open the phase III component of protocol l 06–31 which will randomize patients diagnosed with bone metastases between SBRT and other forms of external beam radiation therapy. While the protocol does stipulate [22] that for those randomized to the conventional treatment arm “field arrangements to treat the target lesion may be chosen at the discretion of the treating radiation oncologist” the study goes on to provide detailed suggestions for treating the spine via 2-field approaches (AP-PA for thoraco-lumbar spine; lateral beams for cervical spine) with only cursory mention of the possibility of using oblique beams.

The absence of rigorous comparisons of portal selection is also evident in reference textbooks [13] as well as the recent evidence-based ASTRO guidelines on palliative radiotherapy for bone metastases [6] which provide extensive information on dose volume relationships but fail to bring up the matter of optimal field arrangements when conventional irradiation is employed. The reality is that 3D conformal RT is already the standard procedure for irradiation in many institutions worldwide. However, even in the most recently published update of the International Consensus on palliative RT endpoints for future clinical trials in bone metastases opinion was split between prescribing the dose to the mid-vertebral body or anterior vertebral body for a single direct field and the guidelines for conformal radiotherapy and stereotactic radiotherapy were subsumed under the rubric of “future research areas” [23]. Ironically, the same guidelines devote extensive space towards consideration of SBRT techniques despite the fact that the use of the latter is not yet justifiable with level 1 evidence. Since the authors of these guidelines do not yet consider SBRT to be a standard of care, we believe that data must still be collected regarding the merits of modern as well as conventional beam arrangements.

It is troubling to reflect on the current reality of extremes. Today’s clinician seems to be left with polarized options for treating bone metastases of the spine. Specifically, physicians can avail themselves of the most modern variants of SBRT; however, there are certainly many palliative cases which do not require this level of sophistication. Conversely, alternative options that are documented in the literature include the most rudimentary radiation techniques (i.e. Posterior beams or combination of AP and PA fields). The rationale for presenting our results is to re-orient the clinician to an intermediate level of sophistication in the palliative approach to this entity.

## Conclusions

In summary, 3D conformal radiation treatment of lumbar vertebral metastases was significantly better in term of bowel and spinal cord exposure when compared to AP-PA and PA techniques. The routine deployment of stereotactic body radiation therapy and intensity modulated radiation therapy for management of bone metastases may represent an unnecessary expenditure of resources. Clinicians should therefore continue to avail themselves of 3D conformal approaches as dictated by the complexity of the clinical case being evaluated.

## Abbreviations

AP-PA: Anterior- posterior- posterior-anterior; 3D: Three dimensional; IMRT: Intensity modulated radiation therapy; 2D: Two dimensional; ICRU: the International Commission on Radiation Units and Measurements; GTV: Gross tumor volume; CTV: Clinical target volume; PTV: Planning target volume.

## Competing interests

The authors declare that they have no competing interests.

## Authors’ contributions

VS, HT and BC designed the study; VS, HT, NS and DS collected and analyzed data; VS and BC wrote the manuscript. All authors read and approved the final manuscript.

## Supplementary Material

Additional file 1: Table S1 Critical organs radiation exposure. The table depicts an exposure of kidneys, spinal cord and small bowel to radiation. Click here for file
