# Supplementary material for: The advantage of 3D conformal treatment of lumbar spine metastases in comparison to traditional PA or AP-PA techniques: restoring an intermediate niche of therapeutic sophistication
Source: Radiat Oncol. 2013 Feb 12;8:34. doi: 10.1186/1748-717X-8-34 (PMC3599142; doi:10.1186/1748-717X-8-34)
Supplement: Additional file 1: Table S1 — Critical organs radiation exposure. The table depicts an exposure of kidneys, spinal cord and small bowel to radiation. [file 1748-717X-8-34-S1.pdf]

	Bowel V15 (%)	
	6.6967(3 D)	
**p<.0001**	39.7800 (AP-PA)	p<.0001
**p=0.685**	37.3392 (PA)	

	Bowel (mean dose -Gy)	
	8.7 (3D)	
**p=.005**	11,6 (AP-PA)	p=.88
**p=.0003**	9.15 (PA)	

	Kidney (mean dose Gy)	
	9.6 (3D)	
**p=.0003**	4.1 (AP-PA)	p=.0007
**p=.023**	4.6 (PA)	

	Spinal cord (maximal dose Gy)	
	30.56 (3D)	
**p<.0001**	33.12 (AP-PA)	p<.0001
**P<.0001**	37.72 (PA)	
